# Sudden cardiac death in younger adults: a comparative analysis of coronary and non-coronary causes

**DOI:** 10.1016/j.ijcha.2026.102009

**Published:** 2026-09-07

**Authors:** Pia Thiesing, Andreas Smit, Hendrik Milting, Nazha Hamdani, Philip Scheene, Moritz Seiffert, Andreas Mügge, Ibrahim El-Battrawy, Assem Aweimer

**Affiliations:** a Department of Cardiology and Angiology, Bergmannsheil University Hospital, Ruhr University Bochum, Bochum, Germany; b Erich and Hanna Klessmann-Institute for Cardiovascular Research and Development, Heart- and Diabetes Center NRW, University Hospital of the Ruhr University Bochum, Germany; c Department of Cellular and Translational Physiology, Institute of Physiology, Ruhr University Bochum, Bochum, Germany; d Institut für Forschung und Lehre (IFL), Molecular and Experimental Cardiology, Ruhr University Bochum, Bochum, Germany; e Department of Electrophysiology, Alfried Krupp Hospital, Essen, Germany

**Keywords:** Sudden cardiac death, Coronary, Non-coronary

## Abstract

**Aims:**

We aimed to characterize etiologies and outcomes of patients aged 16–65 years after resuscitated sudden cardiac death (SCD), with particular emphasis on non-coronary and idiopathic cases.

**Methods:**

This retrospective single-center study screened admissions from 2010 to 2021 using ICD-10 codes followed by manual chart review. Patients were classified as having coronary, non-coronary, or unclassified etiology. Follow-up of hospital survivors included chart review, structured interviews, and device interrogation where available.

**Results:**

Of 232 patients meeting the initial inclusion criteria, 187 had sufficient diagnostic information for etiological classification: 139 had coronary and 48 non-coronary causes, while 45 remained unclassified because early death or incomplete diagnostic information precluded reliable assignment. In the classified cohort, 136 patients (72.7%) experienced out-of-hospital cardiac arrest. Coronary artery disease accounted for 94.2% of coronary cases. In the non-coronary group, 54.2% remained idiopathic; identified causes included cardiomyopathies (29.2%), myocarditis (8.3%), and channelopathies (6.3%). CPR duration <10 min was associated with lower in-hospital mortality, whereas duration ≥60 min was associated with higher mortality. Of 118 hospital survivors, structured follow-up was completed in 74 patients (median 77 months). ICD implantation was more frequent in non-coronary than coronary cases (68.8% vs. 18.7%). Among 47 patients with device follow-up, appropriate ICD shocks occurred in 23.1% and 19.0%, respectively.

**Conclusions:**

Non-coronary and idiopathic etiologies represent a substantial proportion of SCD in younger adults. The high proportion of unexplained cases supports standardized post-arrest evaluation, including multimodality imaging, inherited arrhythmia assessment, genetic testing, family screening, and longitudinal expert review. Recurrent ventricular arrhythmias in both groups support ICD-based secondary prevention in selected survivors.

## Graphical abstract

**Figure:**
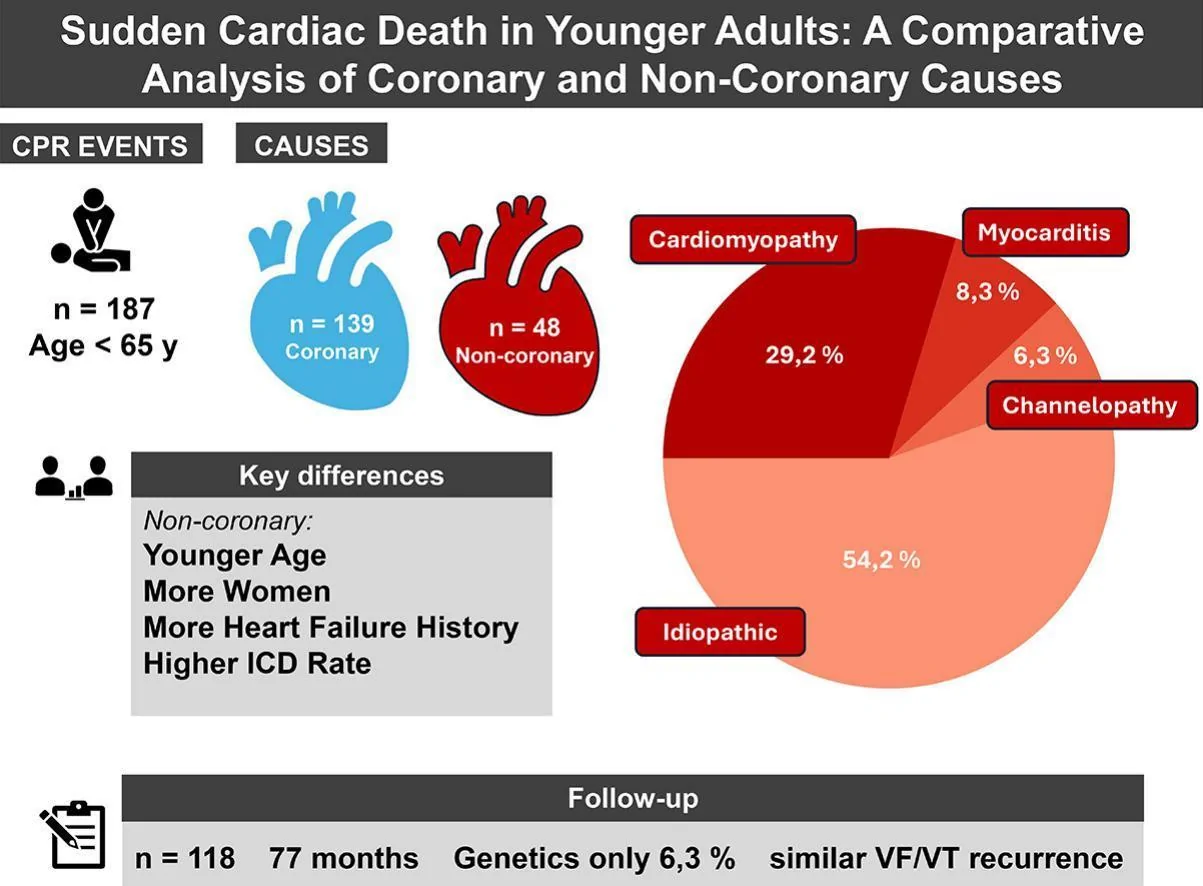

## 1 Introduction

Sudden cardiac death (SCD) is a major global health issue, accounting for an estimated 4–5 million deaths annually [1]. In 2023, the “Lancet Commission to reduce the global burden of sudden cardiac death” emphasized the urgent need to improve survival and long-term outcomes in SCD patients. While high-profile cases, especially among young athletes, often draw public attention, the broader societal impact of SCD, including mortality, disability, and psychological trauma, is substantial and far-reaching.

According to the recent European Society of Cardiology (ESC) guidelines, SCD is defined as sudden natural death presumed to be of cardiac cause, occurring within one hour of symptom onset in witnessed cases, or within 24 h of last being seen alive in unwitnessed cases [2]. Slight variations in this definition across studies and institutions contribute to the difficulty in comparing epidemiological data [3], [4], [5].

The burden of SCD in terms of years of potential life lost exceeds that of many other leading causes of death [6]. A 2014 U.S. study reported an age-adjusted incidence of 54–66 per 100.000 inhabitants, whereas a European multicenter registry reported rates between 36.8 and 39.7 per 100,000 per year [6]. However, incidence rates vary widely depending on the study population and are influenced by age, sex, and underlying health conditions [7], [8], [9], [10].

While the EuReCa ONE study (2016) examined out of hospital cardiac arrest (OHCA) across 27 European countries and demonstrated significant inter-country variability in cardiopulmonary resuscitation (CPR) outcomes, it primarily focused on resuscitation metrics rather than comprehensive data on SCD, especially in unwitnessed cases. Therefore, although informative regarding preclinical emergency response, EuReCa does not fully capture the epidemiology of SCD, particularly in younger individuals or those with unexplained causes [11].

Coronary artery disease (CAD) remains the predominant cause of SCD, accounting for approximately 70–80% of cases. Cardiomyopathies contribute to around 10%. Yet, a considerable proportion of SCD cases occur in individuals without structural heart disease. Among these, primary electrical disorders—such as Long or Short QT syndrome, Brugada syndrome, or early repolarization syndromes—are sometimes identified shortly after the event. This condition has been observed to disproportionately affect younger individuals, often in idiopathic cases, who typically lack traditional cardiovascular risk factors [12], [13], [14], [15], [16], [17].

Multiple studies suggest that this population is frequently underdiagnosed due to the limited application of advanced diagnostic methods such as genetic testing or targeted electrophysiological evaluation. This diagnostic gap underscores the need for more refined strategies to identify inherited arrhythmia syndromes and other concealed etiologies in the young.

Despite its clinical relevance, large-scale studies focusing specifically on SCD in young individuals without CAD, particularly in Germany, are scarce.

In this monocentric study, we aimed to characterize the causes and clinical outcomes of SCD in a young population in the Ruhr region of Germany. Special emphasis was placed on distinguishing between coronary and non-coronary etiologies, with a focus on idiopathic cases. We also evaluated long-term outcomes to contribute to the limited data available on this specific and often-overlooked population.

## 2 Methods

### 2.1 Study design and patient inclusion

This retrospective, monocentric study was conducted at the University Hospital Bergmannsheil in Bochum, Germany. The primary objective was to investigate demographic characteristics, underlying causes, and outcomes of sudden cardiac death (SCD) in a young patient population.

We screened all admissions between January 2010 and December 2021 using the hospital's internal digital registry. The ICD-10 codes I47.2 (ventricular tachycardia), I49.0 (ventricular fibrillation and flutter), I46.- (cardiac arrest/survived sudden cardiac death), and R57.0 (cardiogenic shock) were used as a broad and sensitive screening strategy to identify potential cases of resuscitated cardiac arrest. Importantly, these codes were not used as stand-alone inclusion criteria. All potentially eligible cases were subsequently reviewed manually, and patients were only included if the medical records documented a cardiac arrest or sudden cardiac death event requiring resuscitation, defibrillation, or advanced life support.

The classification as OHCA or in-hospital cardiac arrest (IHCA) was based on documentation from emergency medical services, emergency department records, intensive care records, and hospital charts. Cases identified through VT or cardiogenic shock codes were excluded if no cardiac arrest, CPR, defibrillation, or comparable resuscitation scenario was documented.

Patients aged 16–65 years at the time of the index event were eligible for inclusion. We excluded patients with clearly non-cardiac causes of arrest, including trauma, intoxication, severe metabolic derangements, or other non-cardiac conditions. Resuscitation events occurring peri- or post-interventionally, for example during surgery or cardiac catheterization, were also excluded. Duplicate records and cases with insufficient documentation were removed.

Patients who died shortly after presentation without sufficient diagnostic information for etiological classification were not included in the comparative coronary versus non-coronary analyses. These patients were retained only for the description of the initial screened SCD cohort where applicable, but were excluded from analyses requiring definite etiological assignment.

**Fig. 1:**
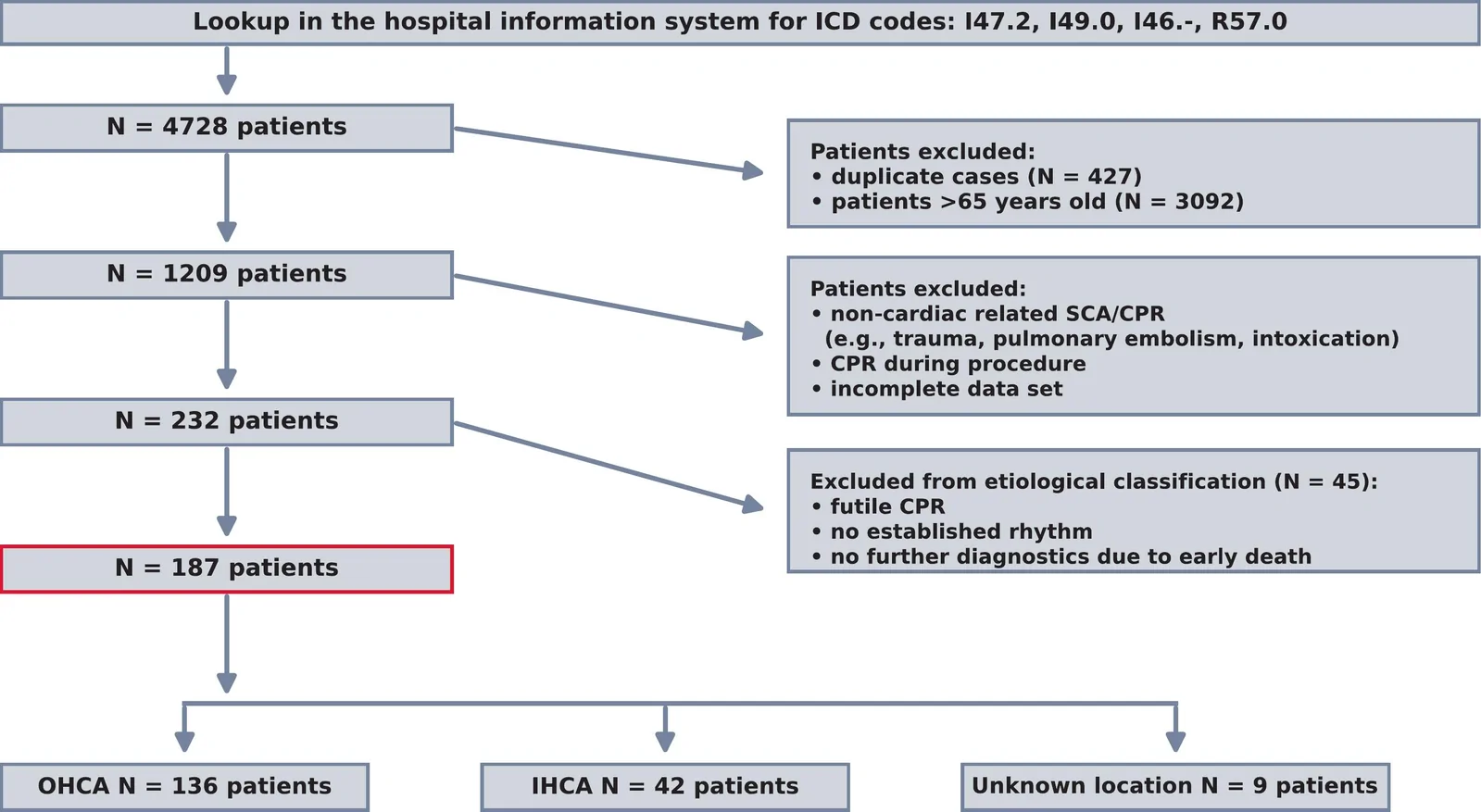
**Schematic flow chart of patient selection.** Stepwise screening and exclusion of patients identified using ICD-10 codes I47.2, I49.0, I46.-, and R57.0. After manual chart review, 232 patients met criteria for the initial SCD cohort, of whom 187 had sufficient diagnostic information for etiological classification.

For clarity, patient numbers are reported according to the respective analysis set. The initial SCD cohort consisted of 232 patients after screening and exclusion of non-eligible cases. Among these, 187 patients had sufficient diagnostic information for etiological classification and were included in the comparative analysis of coronary and non-coronary causes. This classified cohort comprised 139 patients with coronary etiologies and 48 patients with non-coronary etiologies. The remaining 45 patients could not be assigned to either etiological group because early death or incomplete diagnostic information precluded reliable classification. Long-term follow-up analyses were restricted to hospital survivors with available follow-up data (Fig. 1).

### 2.2 Follow-up and outcome assessment

Of the initial cohort of 232 patients, 114 patients died before hospital discharge. The remaining 118 hospital survivors were considered eligible for follow-up. Structured long-term follow-up could be completed in 74 patients and was performed for a median duration of 77 months (IQR 48.75–108.25).

**Fig. 2:**
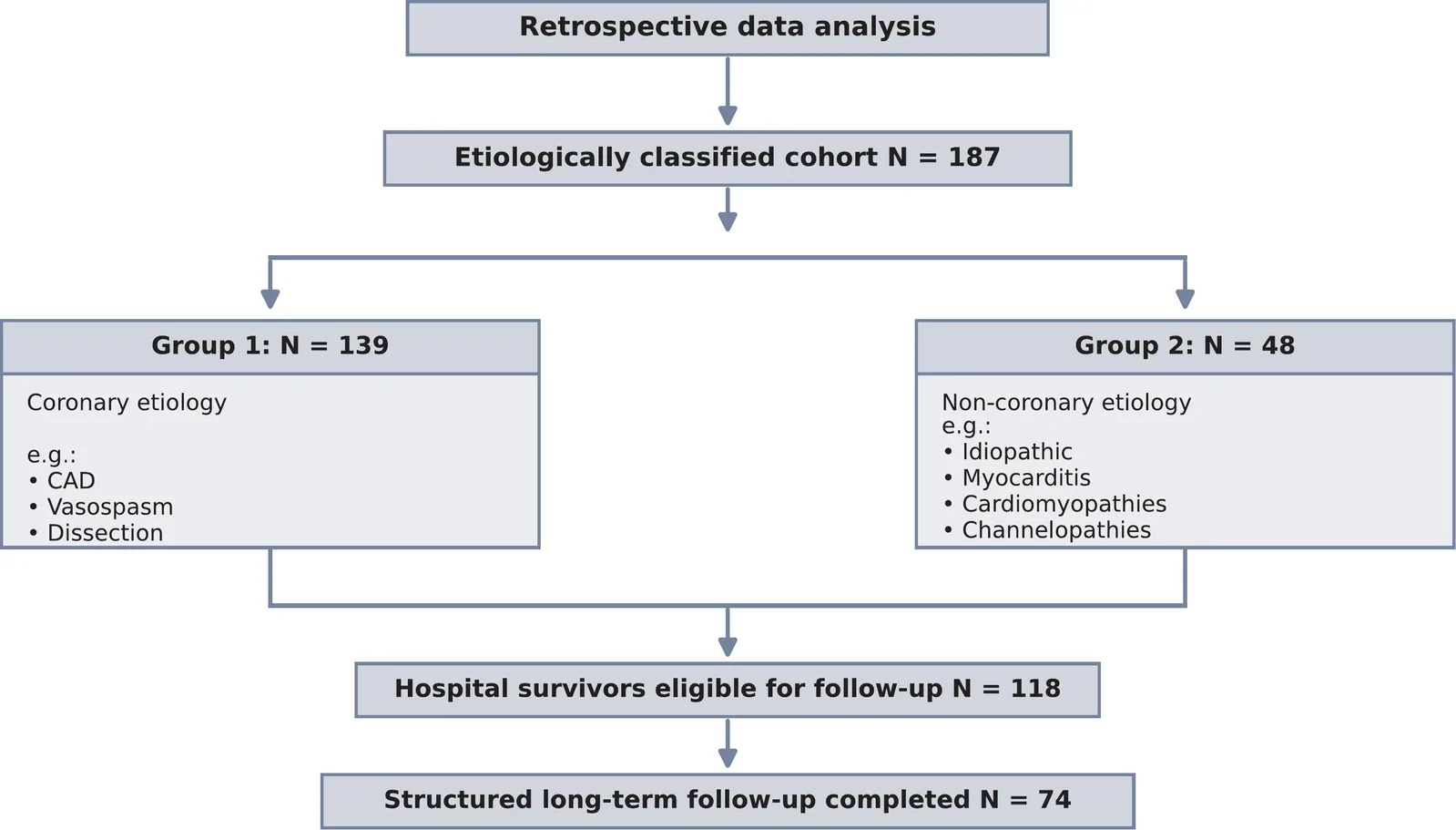
Etiological classification and follow-up flow chart. Patients were assigned to coronary (Group 1) or non-coronary (Group 2) etiologies. Among 118 hospital survivors eligible for follow-up, structured long-term follow-up was completed in 74 patients.

Follow-up data were collected after written informed consent had been obtained and in accordance with data protection regulations. The study was approved by the ethics committee of Ruhr University Bochum (approval number: 22–7466). Follow-up information was obtained from hospital records, structured patient or family interviews, treating general practitioners, cardiologists, and other physicians involved in post-discharge care. In patients with implanted cardiac devices, available ICD interrogation reports from our institution or external cardiology practices were reviewed. Follow-up was completed in March 2023 (Fig. 2).

The main follow-up outcomes were recurrence of sustained ventricular tachyarrhythmias, appropriate ICD therapies including shocks or anti-tachycardia pacing (ATP), and overall survival. Additional outcomes included ICD implantation rates, ICD-related complications, inappropriate ICD therapies, changes in left ventricular ejection fraction (LVEF) at 3–6 months after the index event, and hospital readmissions. Indications for ICD implantation and, where available, reasons for non-implantation were extracted from the medical records.

### 2.3 Statistical analysis

Continuous variables with normal distribution are presented as mean ± standard deviation (SD), whereas non-normally distributed variables are reported as median and interquartile range (IQR). Categorical variables are presented as absolute numbers and percentages. Normality was assessed using the Kolmogorov–Smirnov test. Group comparisons were performed using Student's *t*-test for normally distributed continuous variables, the Mann–Whitney *U* test for non-normally distributed continuous variables, and the χ^2^ test or Fisher's exact test for categorical variables, as appropriate.

Multivariable logistic regression analyses were performed to explore factors associated with in-hospital mortality. Variables with *P* ≤ 0.05 in univariate analysis were considered for inclusion in the multivariable model. Results are reported as odds ratios (ORs) with corresponding *P*-values. These analyses were restricted to patients with sufficient diagnostic and clinical information, particularly the etiologically classified cohort, and should be interpreted as exploratory due to the retrospective design and potential residual confounding.

Analyses of resuscitation-related variables, including bystander CPR, CPR duration, and ECLS use, were interpreted cautiously because of potential confounding by arrest circumstances, no-flow and low-flow times, and initial clinical severity.

All statistical analyses were performed using IBM SPSS Statistics, versions 28.0 and 29.0.2.0. A two-sided *P*-value ≤0.05 was considered statistically significant.

## 3 Results

### 3.1 Study population

Among the 187 etiologically classified patients, 136 (72.7%) experienced out-of-hospital cardiac arrest, 42 (22.5%) had in-hospital cardiac arrest, and arrest location was unclear in 9 (4.8%). (Fig. 1).

Patients were categorized into two etiological groups (Fig. 2):

• Group 1 (*n* = 139): Coronary causes (e.g. coronary artery disease [CAD], vasospasm, acute coronary dissection)

• Group 2 (*n* = 48): Non-coronary causes (e.g. cardiomyopathies, myocarditis, channelopathies, idiopathic cases)

Among patients with coronary etiologies, CAD was the predominant cause, accounting for 94.2% of Group 1 cases. Less frequent coronary-related mechanisms included coronary vasospasm (2.2%), acute coronary dissection (2.2%), and thrombotic occlusion (1.4%). In the non-coronary group, more than half of the patients remained classified as idiopathic despite diagnostic workup (54.2%). Identified non-coronary causes included cardiomyopathies (29.2%), myocarditis (8.3%), channelopathies (6.3%), and valvular disease (2.1%) (Table 1). Unless otherwise specified, comparisons between coronary and non-coronary etiologies refer to the etiologically classified cohort of 187 patients.

**Table 1:** Baseline characteristics and etiological classification of the classified cohort.

Variables	All Patients *N* = 187	Group 1 *N* = 139	Group 2 *N* = 48	*p*-value
Total –– (%)	187 (100)	139 (100)	48 (100)	
Age –– years, median (min-max)	56 (16–65)	56 (34–65)	52 (16–65)	**0.001**
Male –– no. (%)	155 (82.9)	123 (88.5)	32 (66.7)	**< 0.001**
BMI –– kg/m^2^ median (min-max)	26.3 (18.67–70.31)	26.3 (18.67–59.49)	26.23 (19.49–70.31)	0.953
Deceased during hospitalization –– no. (%)	69 (36.9)	55 (39.6)	14 (29.2)	0.227
**Medical history of pre-existing conditions–– No. (%)**				
Smoker active	62 (33.2)	58 (41.7)	4 (8.3)	**<0.001**
Arterial Hypertension	97 (51.9)	76 (54.7)	21 (43.8)	0.241
Diabetes mellitus	38 (20.3)	32 (23)	6 (12.5)	0.147
Dyslipidemia	60 (32.1)	47 (33.8)	13 (27.1)	0.474
Previous myocardial infarction	26 (13.9)	26 (18.7)	0 (0)	**<0.001**
Heart failure	20 (10.7)	10 (7.2)	10 (20.8)	**0.014**
**Further Cardiovascular comorbidity:**	54 (28.9)	37 (26.6)	17 (35.4)	0.27
Valve defect	15 (8)	7 (5)	8 (16.7)	**0.026**
**Any rhythmological disorders** *➔* Atrial fibrillation *➔* Sick-Sinus-Syndrome *	16 (8.6) 16 (8.6) 2 (1.1)	8 (5.8) 8 (5.8) 0 (0)	8 (16.7) 8 (16.8) 2 (4.2)	**0.033**
**AV- Block**	2 (1.1)	1 (0.7)	1 (2.1)	0.449
*➔* I° *➔* II° *➔* III°	1 (0.5) 0 (0) 1 (0.5)	0 (0) 0 (0) 1 (0.7)	1 (2.1) 0 (0) 0 (0)	
Neurological disorders	11 (5.9)	3 (2.2)	8 (16.7)	**0.001**
Familial predisposition	39 (20.9)	27 (19.4)	12 (25)	0.416
Median age of affected relatives at event	60 (37–80)	60 (37–80)	60 (47–76)	0.97
**Diagnosed cause of SCA- no. (%)**				
Idiopathic	26 (13.9)	0 (0)	26 (54.2)	**<0.001**
Coronary heart disease	131 (70.1)	131 (94.2)	0 (0)	**<0.001**
Coronary Vasospasm	3 (1.6)	3 (2.2)	0 (0)	0.571
Thrombotic occlusion	2 (1.1)	2 (1.4)	0 (0)	1.0
Coronary dissection	3 (1.6)	3 (2.2)	0 (0)	0.571
**Cardiomyopathy**	14 (7.5)	0 (0)	14 (29.2)	**< 0.001**
*➔* DCM	5 (2.7)	0 (0)	5 (10.4)	**<0.001**
*➔* HOCM	1 (0.5)	0 (0)	1 (2.1)	0.257
*➔* HNCM	3 (1.6)	0 (0)	3 (6.3)	**0.016**
*➔* Non-compaction	0 (0)	0 (0)	0 (0)	–
*➔* Inflammatory CMP	2 (1.1)	0 (0)	2 (4.2)	0.065
*➔* Secondary CMP	2 (1.1)	0 (0)	2 (4.2)	0.065
*➔* Not defined CMP	1 (0.5)	0 (0)	1 (2.1)	0.257
Myocarditis	4 (2.1)	0 (0)	4 (8.3)	**0.004**
Valvular disease	1 (0.5)	0 (0)	1 (2.1)	**0.257**
**Channelopathies**				
Long QT- Syndrome *➔* Congenital	2 (1.1) ➔1 (0.5)	0 (0)	2 (4.2) ➔1 (2.1)	0.065
Short QT- Syndrome	1 (0.5)	0 (0)	1 (2.1)	**0.0257**

Etiological categories are shown for patients with sufficient diagnostic information for classification (n = 187). Patients without assignable etiology due to early death or incomplete diagnostic information (*n* = 45) are not included in this table. Group 1 = coronary etiology; Group 2 = non-coronary etiology. **ARVC**: Arrhythmogenic Right Ventricular Cardiomyopathy; **AV-block**: Atrioventricular block; **BMI**: Body-Mass-Index; **CMP**: Cardiomyopathy; **CPR:** Cardiopulmonary resuscitation; **DCM**: Dilated cardiomyopathy; **HCM**: Hypertrophic Cardiomyopathy; **HNCM**: Hypertrophic nonobstructive Cardiomyopathy; **HOCM**: Hypertrophic obstructive Cardiomyopathy; **ICU**: intensive care unit; **PAD**: peripheral artery disease; **SCA:** Sudden cardiac arrest; **Secondary CMP:** Myocardial pump failure in highly impaired pump function due to pre-existing muscular dystrophy of the limb-girdle type with cardiac involvement/ CMP in Fallot's tetralogy

* Two patients were diagnosed with sick sinus syndrome and both also presented with atrial fibrillation.

### 3.2 Baseline characteristics and demographics

In the etiologically classified cohort, the median age was 56 years (range: 16–65 years). Patients with coronary etiologies were slightly older than those with non-coronary etiologies, with a median age of 56 years (range: 34–65) in Group 1 and 52 years (range: 16–65) in Group 2. Male sex predominated in both groups, but was more frequent in the coronary group (88.5%) than in the non-coronary group (66.7%). As expected, cardiovascular risk factors, including active smoking, diabetes mellitus, arterial hypertension, and prior myocardial infarction, were more prevalent among patients with coronary causes (Table 1).

### 3.3 Performance of bystander CPR and LVEF at admission

Bystander CPR was documented in 39.6% of all patients, including 37.4% of patients with coronary etiologies and 45.8% of patients with non-coronary etiologies (Table 3). In the remaining cases, no bystander CPR was documented before professional medical assistance. In exploratory multivariable analysis, bystander CPR was not independently associated with in-hospital survival; however, this finding should be interpreted cautiously because information on arrest circumstances, no-flow time, CPR quality, and emergency medical service response intervals was incomplete.

**Fig. 3:**
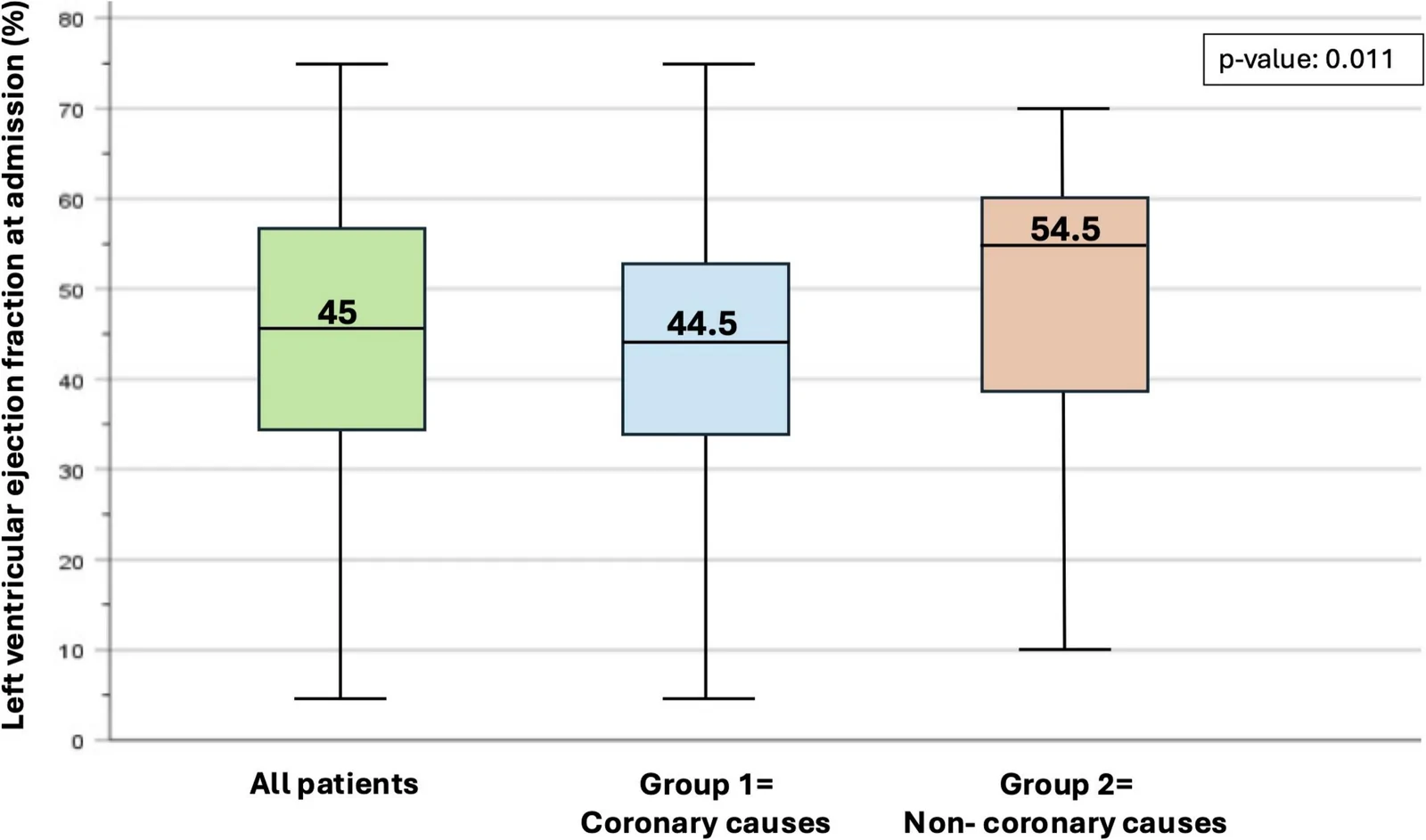
**Left ventricular ejection fraction (LVEF) at admission**.

LVEF at admission varied substantially between individuals, with a median of 44.5% in the coronary group and 54.5% in the non-coronary group (Table 2, Fig. 3). Because early post-arrest LVEF may be influenced by transient myocardial stunning, these values should be interpreted as descriptive admission findings rather than as definitive markers of chronic ventricular function.

**Table 2:** Clinical presentation, diagnostic workup, and in-hospital treatment.

Variables	All Patients *N* = 187	Group 1 *N* = 139	Group 2 *N* = 48	p-value
**Clinical presentation – no. (%)**				
Angina pectoris	26 (13.59)	24 (17.3)	2 (4.2)	**0.028**
NSTEMI	35 (18.7)	31 (22.3)	4 (8.3)	**0.033**
STEMI	76 (40.6)	76 (54.7)	0 (0)	**<0.001**
**Left ventricular ejection fraction at admission (%)**				
Median (min-max)	45 (5–75)	44.5 (5–75)	54.5 (10–70)	**0.011**
Mean (± std.deviat.)	44.12 (±14.48)	42.49 (±14.127)	48.55 (±14.667)	**0.026**
First body temperature (in C°) – Median (min-max)	36.3 (31.8–38.7)	37 (34–38)	35.8 (31.8–36.4)	0.119
**Intra-hospital diagnostics:**				
*➔* Ajmaline	6 (3.2)	0 (0)	6 (12.5)	**<0.001**
*➔* Echocardiography	161 (86.1)	119 (85.6)	42 (87.5)	1.00
*➔* CMR	27 (14.4)	7 (5)	20 (41.7)	**<0.001**
*➔* Exercise ECG	2 (1.1)	1 (0.7)	1 (2.1)	0.449
Electrophysiology study done	5 (2.7)	1 (0.7)	4 (8.3)	**0.016**
Previous EPU	1 (0.5)	1 (0.7)	0 (0)	1.00
Genetics done	5 (2.7)	2 (1.4) *^1^	3 (6.3)	0.108
Coronary angiography	163 (87.2)	126 (90.6) *^2^	37 (77.1)	**0.023**
PCI	112 (59.9)	109 (87.4)	3 (6.3) *^3^	**<0.001**
Bypass surgery planned or recommended	7 (3.7)	7 (5)	0 (0)	0.194
**Treatment approaches during hospital stay:**				
**Targeted temperature management (TTM)**	56 (29.9)	36 (25.9)	20 (41.7)	**0.043**
Time of hypothermia –– hours, median (min-max)	24 (6–25)	24 (7–25)	24 (6–24)	0.549
Lowest achieved temperature –– °C, median (min-max)	33.1 (31.9–36)	33.1 (31.9–35.5)	33.75 (32–36)	0.403
**New ICD- Implantation during index hospitalization**	44 (23.5)	20 (14.4)	24 (50)	**<0.001**
Recommended or planned implantation	8 (4.3)	3 (2.2)	5 (10.4)	**0.028**
Already implanted	7 (3.7)	2 (1.4)	5 (10.4)	**0.013**
If no ICD implantation: Use of WCD	9 (4.8)	4 (2.9)	5 (10.4)	**0.05**
Recorded ventricular tachyarrhythmias using WCD	0 (0)	0 (0)	0 (0)	-
Implantation of pacemaker ➔ Temporary *➔* Permanently	10 (5.3) - 6 (3.2) - 4 (2.1)	4 (2.9) - 4 (2.9) - 0 (0)	6 (12.5) - 2 (4.2) - 4 (8.3)	**0.019** - 0.648 **- 0.004**
**Use of circulatory assist device**	40 (21.4)	35 (25.2)	5 (10.4)	**0.04**

**ECG**: Electrocardiogram; **EPU**: Electrophysiological Study; **ICD**: Implantable Cardioverter-Defibrillator; **CMR:** cardiac magnetic resonance imaging; **NSTEMI**: Non-ST-elevation myocardial infarction; **STEMI**: ST-elevation myocardial infarction; **VF**: Ventricular fibrillation; **VT**: Ventricular tachycardia; **WCD:** wearable cardioverter-defibrillator

*^1^ Two Group 1 patients underwent genetic testing for overlapping DCM and suspected LQTS with Torsades-De-Pointes. One of these patients declined follow-up and no genetic results are available. Nevertheless, he was diagnosed with three-vessel coronary artery disease (CAD), with two vessels treated by PTCA.

*^2^ Twelve patients did not undergo diagnostic PCI due to early death during the initial phase of treatment. Myocardial infarction strongly suspected in the setting of STEMI on arrival or previously known CAD.

*^3^ Three patients in Group 2 underwent PCI - although a stenosis was identified, it was not considered to be the causative lesion.

### 3.4 Clinical presentation and initial monitored heart rhythm

The initial monitored rhythm was predominantly ventricular fibrillation, observed in 75.5% of patients with coronary etiologies and 68.8% of patients with non-coronary etiologies. Asystole was documented in 12.9% and 16.7%, respectively, while pulseless electrical activity and bradyarrhythmic rhythms were less frequent. Sustained ventricular tachycardia was documented more often in the non-coronary group than in the coronary group (12.5% vs. 5.8%) (Table 3).

**Table 3:** Resuscitation characteristics.

Variables	All Patients N = 187	Group 1 N = 139	Group 2 N = 48	p-value
**First monitored rhythm:**				
VF	138 (73.8)	105 (75.5)	33 (68.8)	0.349
VT	14 (7.5)	8 (5.8)	6 (12.5)	0.198
Asystole	26 (13.9)	18 (12.9)	8 (16.7)	0.277
Pulseless electrical activity	4 (2.1)	2 (1.4)	2 (4.2)	0.272
Bradycardia (<40 bpm)	3 (1.6)	2 (1.4)	1 (2.1)	1.00
Missing information	4 (2.1)	4 (2.9)	0 (0)	(0.574)
**Use of defibrillator**	157 (84)	120 (86.3)	37 (77.1)	0.17
Number of shocks delivered –– median (min-max)	3 (1–15)	3 (1–15)	2 (1−12)	**0.034**
**Location at cardiac arrest – no. (%)**				
Home	63 (33.7)	45 (32.4)	18 (37.5)	0.596
Workplace*	27 (14.4)	19 (13.7)	8 (16.7)	0.637
Public place	48 (25.7)	34 (24.5)	14 (29.2)	0.567
Emergency ambulance	6 (3.2)	5 (3.6)	1 (2.1)	1.0
Hospital*	36 (19.3)	28 (20.1)	8 (16.7)	0.676
Unknown	9 (4.8)	8 (5.7)	1 (2.1)	(0.451)
Bystander-witnessed cardiac arrest	161 (86.1)	119 (85.6)	42 (87.5)	1.00
**Bystander CPR performed**	74 (39.6)	52 (37.4)	22 (45.8)	0.276
≤ 10 min	58 (31)	43 (30.9)	15 (31.3)	
> 10 min	11 (5.8)	6 (4.3)	5 (10.4)	
**Time to start CPR among patients with available data**				1.00
≤10 min	139 (74.3)	102 (73.4)	37 (77.1)	
> 10 min	6 (3.2)	5 (3.6)	1 (2.1)	
**Total duration of CPR among patients with available data**				0.076
≤10 min	32 (17.1)	19 (13.7)	13 (27.1)	
> 10 min	125 (66.8)	95 (68.3)	30 (62.5)	
**Pre-hospital duration CPR among patients with available data**				0.116
≤10 min	21 (11.2)	12 (8.6)	9 (18.8)	
> 10 min	103 (55)	77 (55.4)	26 (54.2)	
**Intra- hospital duration CPR among patients with available data**				0.055
≤10 min	19 (10.2)	11 (7.9)	8 (16.7)	
> 10 min	53 (28.3)	44 (31.7)	9 (18.8)	
**CPR in which situation/mechanism?**				
Resting	14 (7.5)	9 (6.5)	5 (10.4)	0.356
Sports	18 (9.6)	12 (8.6)	6 (12.5)	0.409
Non-stressful daily activities	62 (33.2)	47 (33.8)	15 (31.3)	0.859
Working	26 (13.9)	20 (14.4)	6 (12.5)	1.00
Unknown	67 (35.8)	51 (33.1)	16 (31.3)	0.729

**CPR**: Cardiopulmonary resuscitation

*Two patients are counted twice as their workplace -as location of cardiac arrest- was also the hospital

External defibrillation was administered in the majority of patients, with a median number of shocks of 3 in the coronary group and 2 in the non-coronary group. Although an initial shockable rhythm was associated with improved outcome in univariate analysis, this association did not remain statistically significant in multivariable analysis (Table 4).

**Table 4:** Exploratory multivariable logistic regression analysis for in-hospital mortality.

Variable	Multivariable analysis		
	**OR**	**95% CI**	***P* value**
Bystander CPR	0.555	0.23–1.334	0.188
Total duration of CPR in minutes			
≤10 min	0.052	0.006–0.442	**0.007**
≥ 60 min	4.753	1.475–15.314	**0.009**
First monitored rhythm			
Shockable – VF/VT	0.131	0.001–24.729	0.447
Non-shockable – PEA/Asystole	1.034	0.005–211.316	0.990
Treatment chain			
Circulatory assist device	3.789	1.351–10.629	**0.011**

OR > 1 indicates higher odds of in-hospital mortality. Reference categories were no bystander CPR, CPR duration 11–59 min, and absence of circulatory assist device. The model was exploratory and should be interpreted cautiously due to retrospective design and potential residual confounding.

### 3.5 In-hospital diagnostics and therapy

Coronary angiography was performed in 90% of patients with coronary etiologies, and PCI was performed in 87% of patients presenting with acute myocardial infarction. Genetic testing was performed in 5 patients overall, corresponding to 2.7% of the etiologically classified cohort. This included 2 patients in the coronary group and 3 patients in the non-coronary group.

ECLS was used in 25.2% of patients with coronary etiologies and in 10.4% of patients with non-coronary etiologies. In exploratory multivariable analysis, ECLS use was associated with higher in-hospital mortality (OR 3.789, *p* = 0.011). This finding should be interpreted in the context of the retrospective study design and the fact that ECLS was primarily used in patients with refractory cardiac arrest or severe post-arrest circulatory failure. CPR duration was strongly associated with outcome: CPR lasting less than 10 min was associated with improved survival (OR 0.052, *p* = 0.007), whereas CPR duration longer than 60 min was associated with higher mortality (OR 4.753, *p* = 0.009). Targeted temperature management did not show a statistically significant association with survival in this cohort (OR 0.508, *p* = 0.053).

### 3.6 General follow-up data

Of the initial cohort of 232 patients, 114 patients (49.1%) died before hospital discharge. The remaining 118 patients survived to discharge and were considered eligible for follow-up. Structured long-term follow-up was completed in 74 patients. During a median follow-up of 77 months, follow-up mortality was 12.9% in the coronary group and 10.0% in the non-coronary group.

**Fig. 4:**
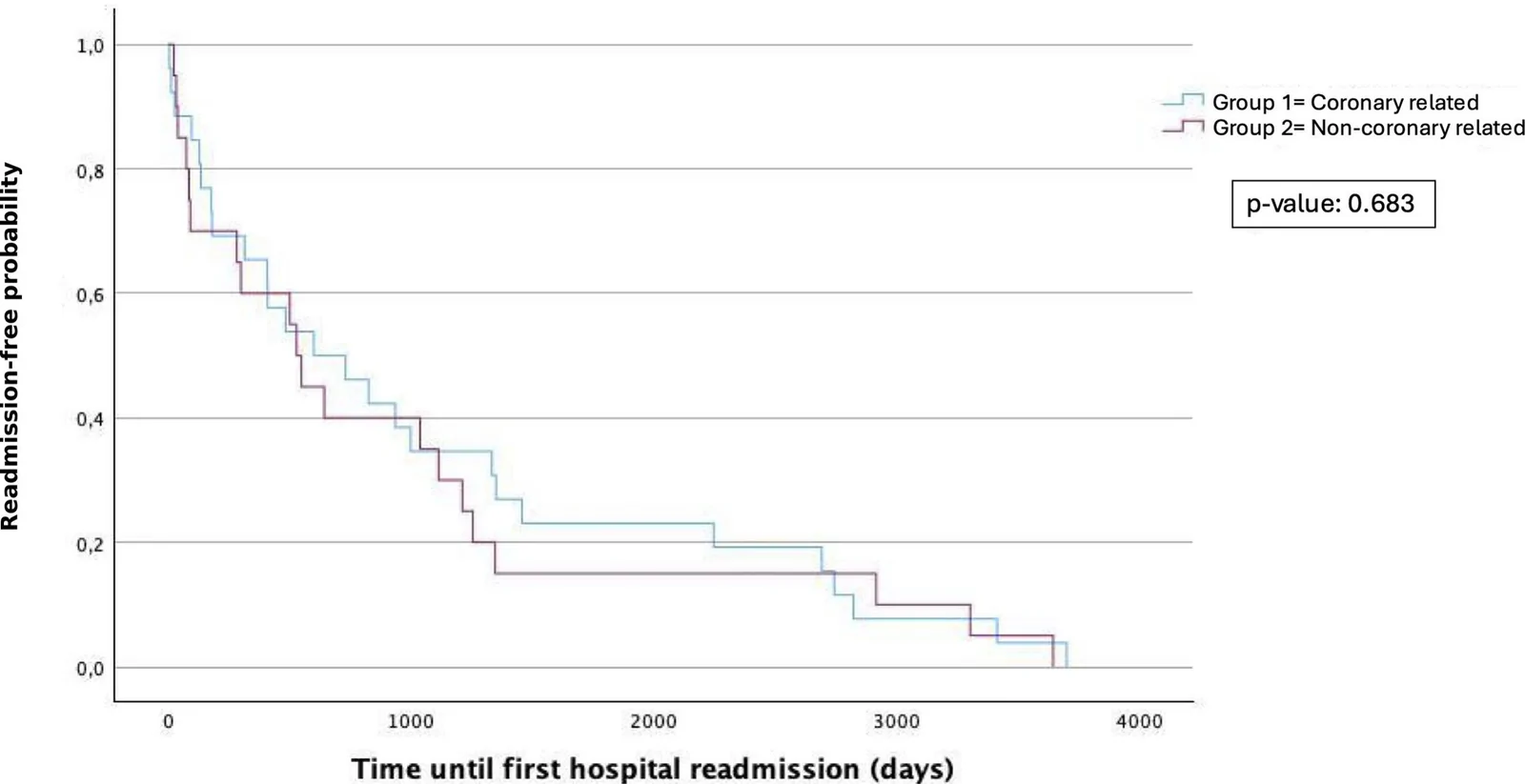
**Time to first hospital readmission during follow-up.** Kaplan–Meier curves show time to first documented hospital readmission among patients with available follow-up data, stratified by coronary and non-coronary etiology. No significant difference was observed between groups (*p* = 0.683).

Hospital readmission was assessed descriptively among patients with available follow-up data. The mean time to first readmission was 1086 days in the coronary group and 949 days in the non-coronary group, with no statistically significant difference between groups (Fig. 4). Because indications for readmission were heterogeneous and were not systematically categorized in all cases, this analysis should be interpreted as a descriptive marker of post-discharge healthcare utilization rather than as a disease-specific endpoint.

Reassessment of LVEF after three to six months showed no relevant difference between groups, with mean values of 55.6% (±11.1) in the coronary group and 55.0% (±11.2) in the non-coronary group (Table 5).

**Table 5:** In-hospital outcomes, long-term follow-up, and ICD therapies.

Variables	All Patients N = 187	Group 1 N = 139	Group 2 N = 48	*p*-value
**In-hospital Outcome – no. (%)**				
Deceased during hospitalization	69 (36.9)	55 (39.6)	14 (29.2)	0.227
Days since admission - mean (standard deviation)	7.77 (14.185)	8.45 (15.670)	5.07 (4.763)	0.474
**Neurological outcome per GOS:**				
1 = Death	69 (36.9)	55 (39.6)	14 (29.2)	0.227
2 = Persistent Vegetative State	2 (1.1)	2 (1.4)	0 (0)	1.0
3 = Severe Disability	5 (2.7)	3 (2.2)	2 (4.2)	0.604
4 = Moderate Disability	8 (4.3)	7 (5)	1 (2.1)	0.336
5 = Good recovery	99 (52.9)	70 (50.4)	29 (60.4)	0.245
Unknown	4 (2.1)	2 (2.2)	2 (4.2)	
**Long-term Outcome**				
Follow-Up data	74/118 (62.7)	43/84 (51.2)	31/34 (91.2)	**<0.001**
Mean Follow Up time in month - Mean (SD)	77.09 (39.491)	76 (39.872)	79 (39.532)	0.108
LVEF (%) after 3–6 month Mean (SD)	55.39 (11.073)	55.59 (11.134)	55.05 (11.223)	0.875
Deceased during FU	11/92 (12)	8/62 (12.9)	3/30 (10)	1.0
- 1-year mortality - 2–5-year mortality Mortality of 5 years and longer	- 4/11 (36.3) - 1/11 (9) 5/11 (45.5)	- 3/8 (37.5) - 1/8 (12.5) 3/8 (37.5)	- 1/3 (33.3) - 0/3 (0) 2/3 (66.7)	1.00 - 1.00 - 0.545
**Cause of death** - Cardiovascular cause - Non-cardiovascular cause of death - Unknown	- 1/11 (9.1) - 1/11 (9.1) - 9/11 (81.8)	- 0/8 (0) - 1/8 (12.5) - 7/8 (87.5)	- 1/3 (33.3) - 0/3 (0) - 2/3 (66.7)	- 0.273 - 1.00 - 0.491
**ICD recipients, n (%)**	59 (31.6)	26 (18.7)	33 (68.8)	**<0.001**
ICD recipients among hospital survivors	54/118 (45.8)	25/84 (29.8)	29/34 (85.3)	**<0.001**
	7 lost to follow-up **47 ICD patients**	4 lost to follow-up **21 ICD patients**	3 lost to follow-up **26 ICD patients**	
**Patients with documented ventricular tachyarrhythmias at Follow-up**	19/47 (40.4)	10/21 (47.6)	9/26 (34.6)	0.390
*➔*sVT*	10/47 (21.3)	6/21 (28.6)	4/26 (15.4)	0.306
*➔* VF*	10/47 (21.3)	3/21 (14.3)	7/26 (26.9)	0.475
*➔* nsVT*	10/47 (21.3)	6/21 (28.6)	4/26 (15.4)	0.306
**Treatment of ventricular tachyarrhythmias at Follow-up**	25/47 (53.2)	11/21 (52.3)	14/26 (53.8)	1.00
Appropriate ICD shock delivery	10/47 (21.3)	4/21 (19)	6/26 (23)	1.00
Inappropriate ICD shock delivery	5/47 (10.6)	1/21 (4.7)	4/26 (15.4)	0.362
Successful ATP	6/47 (12.7)	4/21 (19)	2/26 (7.7)	0.386
Unsuccessful ATP	4/47 (8.5)	2/21 (9.5)	2/26 (7.7)	1.00

**ATP:** Anti-tachycardia pacing; **FU:** Follow-up; **GOS:** Glasgow Outcome Score; **ICD**: Implantable Cardioverter-Defibrillator

*Among the 19 patients with documented ventricular tachyarrhythmias during follow-up, recurrent episodes were observed in some individuals. This resulted in a higher number of reported events than the number of patients experiencing such arrhythmias.

Structured long-term follow-up was completed in 74 of 118 hospital survivors. Vital status during follow-up was available for 92 patients. ICD device follow-up was available in 47 of 54 ICD recipients.

### 3.7 Follow-up after implantation of cardiac devices

ICD implantation for secondary prevention was performed in 26 patients in the coronary group (18.7%) and in 33 patients in the non-coronary group (68.8%). The remaining patients without permanent ICD implantation represented a heterogeneous subgroup, including cases with non-shockable rhythms such as asystole or sinus arrest, suspected conduction disease, myocarditis, severe valvular disease, cardiomyopathy-related decompensation, suspected transient or reversible triggers, and unclear etiologies. However, the exact clinical reasons for non-implantation were not systematically documented in all patients.

**Fig. 5:**
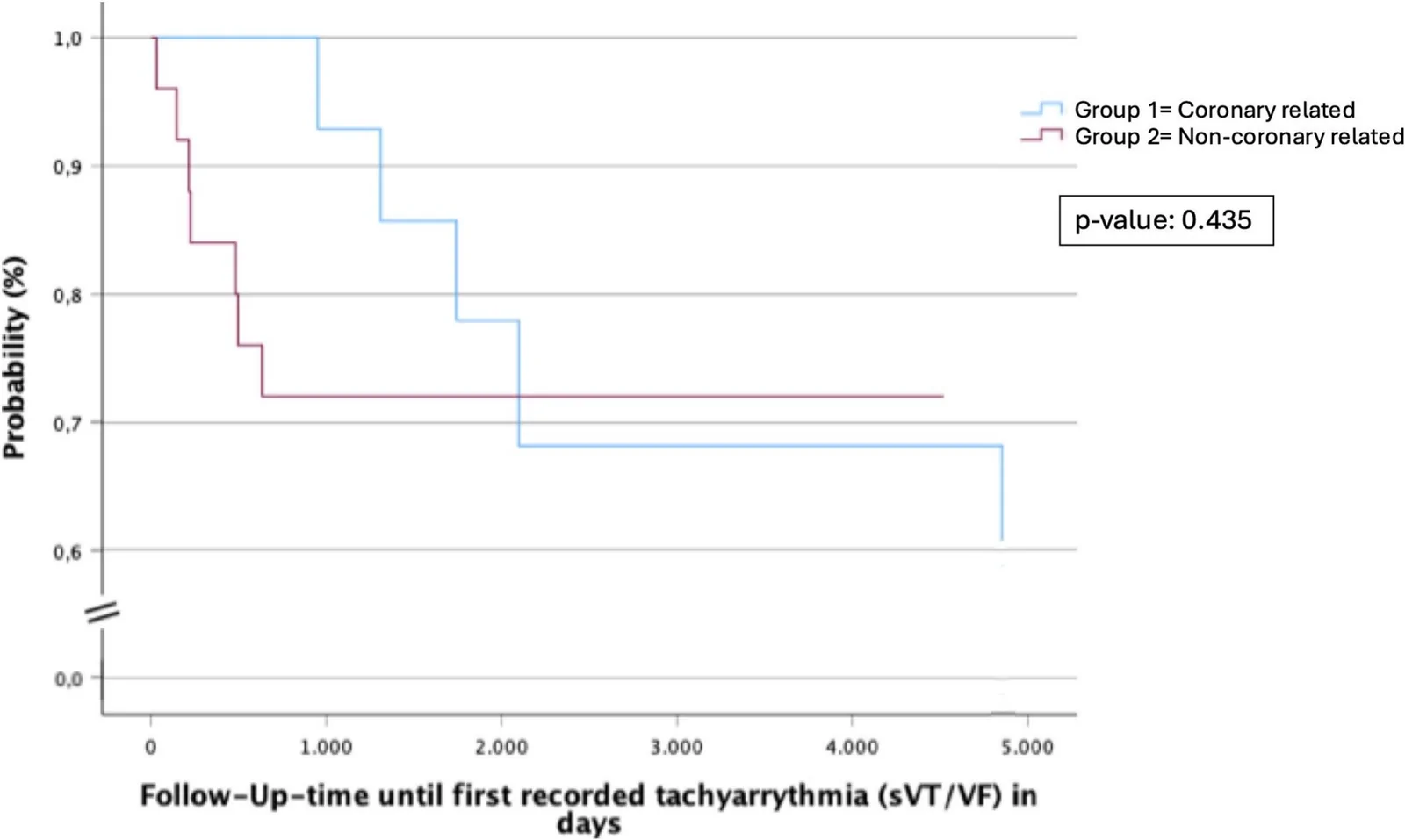
Time to first documented ventricular tachyarrhythmia during ICD follow-up. Kaplan–Meier curves show time to first recorded sustained VT/VF among ICD recipients with available device follow-up, stratified by coronary and non-coronary etiology. No significant difference was observed between groups (*p* = 0.435).

During device follow-up, sustained ventricular tachycardia was documented in 10 patients, including 6 patients in the coronary group and 4 patients in the non-coronary group. Ventricular fibrillation occurred in 10 patients during follow-up, with 7 cases observed in the non-coronary group. Appropriate ICD therapies were observed in 16 patients. ICD shocks were delivered in 4 patients in the coronary group and in 6 patients in the non-coronary group, while successful anti-tachycardia pacing (ATP) was documented in 4 and 2 patients, respectively (Table 5). There was no significant difference in time to first documented ventricular tachyarrhythmia between the two groups (Fig. 5).

## 4 Discussion

This retrospective single-center study provides real-world data on survived sudden cardiac death (SCD) in younger adults aged 16–65 years, with a particular focus on the distinction between coronary and non-coronary etiologies. The main findings are: first, coronary causes remained the most frequent etiology in the overall classified cohort; second, non-coronary causes represented a substantial subgroup and were characterized by a high proportion of idiopathic cases; third, prolonged CPR duration was strongly associated with in-hospital mortality; and fourth, recurrent ventricular tachyarrhythmias requiring appropriate ICD therapy occurred in both coronary and non-coronary SCD survivors during long-term follow-up.

The restriction to patients aged 16–65 years was chosen to reduce the dominance of age-related coronary artery disease and to better capture a population in whom non-ischemic, inherited, inflammatory, or initially unexplained mechanisms are clinically relevant. Although CAD remained the leading cause in our overall cohort, the non-coronary group was younger, included a higher proportion of women, and showed fewer traditional cardiovascular risk factors. These findings are consistent with previous population-based studies showing that the etiological spectrum of SCD shifts away from acute myocardial infarction toward cardiomyopathies, channelopathies, myocarditis, and unexplained arrhythmic death in younger individuals [18].

Importantly, a significant proportion (54,2%) of cases remained without a definitive etiology. In our cohort, 26 cases were initially classified as idiopathic. Upon re-evaluation, we reclassified two of these based on subtle diagnostic findings, underscoring the diagnostic complexity and the value of systematic re-assessment in this population [19]. Recent evidence highlights the diagnostic ambiguity that often surrounds survivors of sudden cardiac arrest without apparent structural heart disease. A study by Merghani et al. revealed that at the time of hospital discharge, only 31% of patients with presumed primary heart rhythm disturbances and no structural abnormalities had a definitive diagnosis. Notably, an additional third of cases were reclassified during extended follow-up through repeated expert review, demonstrating the evolving nature of diagnosis in this subgroup [20]. These findings support the need for prolonged and structured reevaluation in initially idiopathic cases and underscore the limitations of standard in-hospital diagnostics.

A particularly important finding of our study is the high proportion of idiopathic cases within the non-coronary group. More than half of the patients with non-coronary SCD remained without a definitive diagnosis despite clinical workup. This observation is clinically relevant because “idiopathic” ventricular fibrillation is a diagnosis of exclusion and may reflect incomplete phenotyping rather than true absence of underlying disease [19]. Previous studies have shown that repeated expert review and long-term follow-up can lead to diagnostic reclassification in a substantial proportion of initially unexplained cardiac arrest survivors [20]. In line with these observations, re-evaluation in our cohort allowed reclassification of two patients initially considered idiopathic, supporting the concept that diagnostic assessment after unexplained SCD should be regarded as a longitudinal process rather than a single in-hospital evaluation.

Our data also illustrate important diagnostic gaps in real-world post-arrest care. Genetic testing was performed in only 5 patients overall (2.7%), including 3 patients in the non-coronary group. Similarly, advanced phenotyping was not applied uniformly throughout the study period. Cardiac MRI was performed more frequently in the non-coronary group (41,7%) than in the overall cohort (14,4%), but its use was not systematic and likely increased over time with improved availability and growing awareness of its value in unexplained cardiac arrest. Provocative testing for concealed channelopathies, structured family screening, repeat imaging, and formal multidisciplinary inherited arrhythmia review were not implemented as standardized protocols during the entire inclusion period. These limitations likely contributed to the high proportion of idiopathic cases and should be considered when interpreting the etiological distribution [2], [21].

In patients with coronary angiography revealing no significant obstruction, the entity of myocardial infarction with non-obstructive coronary arteries (MINOCA) becomes relevant. Our cohort included 8 such cases (5.8% of all coronary-related presentations), with diagnoses including coronary vasospasm, thrombotic occlusion, and spontaneous coronary artery dissection (SCAD). These findings are consistent with prior reports estimating MINOCA prevalence at 5–15% [22], [23], [24], [25]. However, our real-world incidence of SCAD (2.2%) is higher than that reported in previous registries, which may reflect improvements in in vivo diagnostic techniques or selection bias inherent to survivor cohorts [26], [27], [28], [29]. Importantly, the proportion of coronary etiologies observed in our cohort should not be interpreted as reflecting the overall prevalence of CAD-related SCA in this age group. Our study included a selected hospital-based population of resuscitated patients with sufficient diagnostic information for etiological classification. Population-based data by Waldmann et al. emphasize that CAD remains a major cause of sudden cardiac arrest even among younger adults. Accordingly, the overall burden of CAD-related SCA in this age group is likely higher than suggested by our selected cohort. [30]

The clinical implications of MINOCA and ACS-NNOCA in the context of SCD are still evolving. While the absolute prevalence remains modest, the diverse pathophysiological mechanisms suggest that a one-size-fits-all approach to secondary prevention may be inadequate. In selected patients, intracoronary imaging or vasospasm testing may reveal etiologies not apparent through routine angiography [31].

Our study reaffirms the value of ICDs for secondary prevention in SCD survivors. Notably, 23% of patients with non-coronary SCD who received an ICD experienced appropriate shock therapy, suggesting a relevant risk of recurrent malignant ventricular arrhythmias in this subgroup. The markedly higher ICD implantation rate in the non-coronary group likely reflects the greater proportion of patients without an immediately reversible ischemic trigger after the index event. Nevertheless, not all patients with non-coronary etiologies received a permanent ICD. Patients without permanent ICD implantation formed a clinically heterogeneous subgroup, including individuals with non-shockable rhythms, suspected conduction disease, myocarditis, severe valvular disease, cardiomyopathy-related decompensation, suspected transient or reversible triggers, and unclear etiologies. Because the exact clinical reasons for withholding ICD implantation were not systematically documented in all cases, no firm conclusions can be drawn regarding guideline adherence or individual decision-making. This limitation is inherent to the retrospective design and has to be considered when interpreting ICD implantation rates. Moreover, the long inclusion period from 2010 to 2021 encompassed evolving guideline recommendations and clinical practice regarding secondary-prevention ICD therapy, which may have introduced temporal heterogeneity in ICD decision-making. Inappropriate shocks were observed in 4–15% of patients, a rate comparable to that reported in larger ICD cohorts such as MADIT II [32], [33], [34]. Nevertheless, inappropriate shocks remain clinically relevant due to their psychological impact and their association with increased mortality [32], [33], [35].

CPR duration was one of the strongest predictors of in-hospital outcome in our cohort. Patients who underwent CPR for less than 10 min had significantly better survival, whereas CPR durations beyond 60 min were strongly associated with increased mortality. These findings are consistent with ERC guideline recommendations emphasizing the importance of early return of spontaneous circulation and with large cohort studies showing that prolonged resuscitation is associated with poorer survival and neurological outcomes in both out-of-hospital and in-hospital cardiac arrest [36], [37], [38], [39], [40], [41], [42]. However, our retrospective data do not allow detailed assessment of no-flow time, CPR quality, EMS response intervals, or arrest circumstances. Therefore, resuscitation-related variables, including bystander CPR, should be interpreted cautiously and not as evidence of absent benefit. ECLS use was associated with higher in-hospital mortality in our analysis. This finding should not be interpreted as evidence of harm from ECLS, but rather as a likely reflection of confounding by indication, since ECLS was primarily used in patients with refractory cardiac arrest or severe post-arrest circulatory failure. The present study was not designed to evaluate the efficacy of ECLS, and no conclusions regarding patient selection or treatment benefit can be drawn from these data. Neurological outcome at hospital discharge was favorable in a substantial proportion of the classified cohort, with 52.9% achieving good recovery. Nevertheless, 8.1% had moderate or severe disability or a persistent vegetative state. As detailed neurocognitive and quality-of-life assessments were not available, the long-term neurological burden may have been underestimated. In our cohort, 18 patients (9.6%) experienced cardiac arrest during sports activity. Sports-related SCA should not be regarded as a phenomenon restricted to young competitive athletes; contemporary registry data indicate that most sports-related events occur in recreational athletes, often in middle age. Because detailed information on type and intensity of sports or competitive status was not systematically available in our retrospective dataset, these cases could not be characterized further [43].

### 4.1 Diagnostic gaps and limitations

Several limitations should be acknowledged. First, the retrospective single-center design limits causal interpretation and introduces potential selection bias. Although a broad ICD-10 screening strategy followed by manual chart review was used, complete case ascertainment cannot be guaranteed because miscoded or uncoded cardiac arrest cases may have been missed. Furthermore, despite exclusion of patients with an evident non-cardiac cause of arrest, occult non-cardiac etiologies cannot be completely excluded in cases initially presumed to be cardiac. Previous population-based data in younger patients have demonstrated a relevant contribution of non-cardiac causes among cases undergoing evaluation for presumed SCA, particularly neurological etiologies. This represents an additional potential source of misclassification in retrospective cohorts such as ours [44]. Second, 45 patients from the initial cohort could not be assigned to coronary or non-coronary etiologies because early death or insufficient diagnostic information precluded reliable classification. This introduces survivor bias and may have affected the observed distribution of etiologies, particularly the proportion of idiopathic and non-coronary cases. Third, diagnostic evaluation was not standardized across the entire study period. Genetic testing, cardiac MRI, provocative testing, family screening, repeat imaging, and multidisciplinary inherited arrhythmia review were performed selectively rather than systematically. Consequently, some cases classified as idiopathic may have represented concealed cardiomyopathies, channelopathies, inflammatory disease, or other diagnoses that could have been identified with contemporary standardized protocols. Family screening is particularly important in patients with unexplained or potentially inherited causes of SCA. Systematic evaluation of first-degree relatives may identify previously unrecognized cardiomyopathies or inherited arrhythmia syndromes and thereby extend the diagnostic and preventive benefit beyond the index patient. Current ESC recommendations therefore support structured clinical evaluation of first-degree relatives and, where appropriate, genetic counselling and cascade testing [2]. Fourth, reasons for ICD non-implantation were not systematically documented in all patients, limiting interpretation of device therapy rates. Finally, analyses of bystander CPR, ECLS, and other resuscitation-related variables are subject to confounding by arrest circumstances, initial rhythm, low-flow time, and clinical severity.

## 5 Conclusion

In conclusion, this study shows that among SCD survivors aged 16–65 years, non-coronary etiologies represent a clinically important subgroup and are frequently associated with persistent diagnostic uncertainty. More than half of the non-coronary cases remained idiopathic after routine workup, highlighting the need for structured post-arrest evaluation, including multimodality imaging, inherited arrhythmia assessment, genetic testing, family screening, and longitudinal expert review. Although CAD remains an important cause of SCD in this age range, unexplained and non-coronary mechanisms require greater diagnostic attention. Recurrent ventricular arrhythmias during follow-up support the role of ICD-based secondary prevention in selected survivors, while larger prospective registries are needed to define standardized diagnostic and therapeutic pathways.

## Acknowledgement and funding

No funding was provided, we thank all study participants.

Boxplots display median, interquartile range, and total range of admission LVEF values in the etiologically classified cohort and in subgroups with coronary (Group 1) and non-coronary (Group 2) etiologies.

## CRediT authorship contribution statement

**Pia Thiesing:** Writing – original draft, Formal analysis, Data curation. **Andreas Smit:** Resources, Formal analysis, Data curation. **Hendrik Milting:** Supervision, Methodology, Investigation. **Nazha Hamdani:** Validation, Supervision. **Philip Scheene:** Validation, Investigation. **Moritz Seiffert:** Software, Resources. **Andreas Mügge:** Validation, Supervision, Resources. **Ibrahim El-Battrawy:** Writing – review & editing, Validation, Conceptualization. **Assem Aweimer:** Writing – review & editing.

## Declaration of competing interest

None declared.

## Data Availability

The datasets used for the analysis in the current study and source code for evaluation are available from the corresponding author upon reasonable request.
